# Paediatric Asthma and the Microbiome: A Systematic Review

**DOI:** 10.3390/microorganisms11040939

**Published:** 2023-04-03

**Authors:** Marwh G. Aldriwesh, Abrar M. Al-Mutairi, Azzah S. Alharbi, Hassan Y. Aljohani, Nabeel A. Alzahrani, Reham Ajina, Abdullah M. Alanazi

**Affiliations:** 1 Department of Clinical Laboratory Sciences, College of Applied Medical Sciences, King Saud bin Abdulaziz University for Health Sciences, Riyadh 11481, Saudi Arabia; 2 King Abdullah International Medical Research Center, Riyadh 11481, Saudi Arabia; 3 Ministry of the National Guard-Health Affairs, Riyadh 11426, Saudi Arabia; 4 Research Unit, College of Applied Medical Sciences, King Saud bin Abdulaziz University for Health Sciences, Riyadh 11481, Saudi Arabia; 5 Department of Medical Microbiology and Parasitology, Faculty of Medicine, Jeddah 21362, Saudi Arabia; 6 Special Infectious Agents Unit, King Fahd Medical Research Center, King Abdulaziz University, Jeddah 21362, Saudi Arabia; 7 Department of Respiratory Therapy, College of Applied Medical Sciences, King Saud bin Abdulaziz University for Health Sciences, Riyadh 11481, Saudi Arabia

**Keywords:** asthma, children, dysbiosis, gut microbiome, immunity, lung microbiome

## Abstract

Evidence from the literature suggests an association between the microbiome and asthma development. Here, we aimed to identify the current evidence for the association between asthma and the upper airway, lower airway and/or the gut microbiome. An electronic systemic search of PubMed, EBSCO, Science Direct and Web of Science was conducted until February 2022 to identify the eligible studies. The Newcastle–Ottawa Scale and the Systematic Review Centre for Laboratory Animal Experimentation risk of the bias tools were used to assess quality of included studies. Twenty-five studies met the inclusion criteria. Proteobacteria and Firmicutes were identified as being significantly higher in the asthmatic children compared with the healthy controls. The high relative abundance of *Veillonella*, *Prevotella* and *Haemophilus* in the microbiome of the upper airway in early infancy was associated with a higher risk of asthma development later in life. The gut microbiome analyses indicated that a high relative abundance of *Clostridium* in early childhood might be associated with asthma development later in life. The findings reported here serve as potential microbiome signatures associated with the increased risk of asthma development. There is a need for large longitudinal studies to further identify high-risk infants, which will help in design strategies and prevention mechanisms to avoid asthma early in life.

## 1. Introduction

Asthma is a chronic inflammatory disease that affects the respiratory system and leads to significant morbidity and mortality [1]. Individuals suffering from asthma exhibit an array of symptoms, from wheezing and coughing to chest tightness and shortness of breath [2]. These manifestations vary in time of onset and intensity between asthmatic patients [2]. The common triggers that may lead to asthma exacerbation include, but are not limited to, viral respiratory infections, air pollution, tobacco smoke and exercise [3]. Allergies, genetics, respiratory infections during infancy and environmental features are risk factors for asthma development [3]. However, the exact aetiology of asthma is not well understood.

Evidence from the literature suggests that there is an association between the human microbiome and the development of asthma [4]. Both human studies and studies performed on experimental animal models have linked the dysbiosis of the early-life gut microbiome to a greater risk for the development of asthma in individuals who are genetically susceptible to this disease [4,5,6,7]. The gut microbiome has been shown to regulate the immune responses associated with chronic inflammatory diseases in humans and animal models [5,8]. The establishment of the human gut microbiome starts at birth and is influenced by many factors, including the mode of delivery, antibiotic use and the feeding method [9]. The human gut microbiome consists of not only bacteria but also fungi, protozoa and viruses [10]. The microbiome is dynamic and withstands changes due to age, dietary modifications and environmental and medical interventions, such as the use of antimicrobial agents, throughout an individual’s lifetime [10]. While most of the normal gut microbiome is composed of the phyla Firmicutes and Bacteroidetes, the less common phyla are Actinobacteria, Proteobacteria, Fusobacteria and Verrucomicrobia [10,11]. The most prevalent genera in the normal gut microbiome are *Bacteroides*, *Faecalibacterium* and *Bifidobacterium* [10,11]. The healthy fungal gut microbiome consists mainly of *Saccharomyces cerevisiae*, *Malassezia restricta* and *Candida albicans* [12].

On the other hand, data on the relationship between the lung microbiome and asthma remain limited. This is mainly due to the difficulty of sampling and the long-standing dogma about the lungs being a sterile environment [13]. However, studies have identified the normal lung microbiome, which includes the bacterial genera *Prevotella*, *Streptococcus*, *Veillonella*, *Neisseria*, *Haemophilus* and *Fusobacterium* [13]. Fungal microbiomes in healthy lungs include mainly Ascomycota (*Aspergillus*, *Cladosporium*, *Eremothecium* and *Vanderwaltozyma*) and Microsporidia (*Systenostrema*) [14,15].

In addition, emerging evidence confirms a crosstalk at what is termed the ‘gut–lung axis’, where changes in the gut microbiome may have an impact on the development of lung diseases and vice versa [16,17]. This occurs via the mesenteric lymph nodes, where elements of the microbiome and their metabolites are transported to and from the lungs [18]. Discrepancies in the gut–lung axis are associated with an increased emergence of asthma as well as other acute and chronic respiratory diseases [19].

This systematic review fills the knowledge gaps regarding the association between asthma and the upper airway, lower airway and/or gut microbiome, which has not been specifically addressed previously. In fact, the published systematic reviews have mostly investigated the association between the gut microbiota and asthma or allergic diseases without including the upper and lower airways. In 2018, Zimmerman and colleagues systematically reviewed the intestinal microbiota composition and the development of allergic diseases from birth to 20 years of age [20]. The authors reported that early-life gut microbial exposure indeed has a role in allergic disease development [20]. Melli and colleagues in 2015 examined the early literature (2007–2013) on the link between the gut microbiota and allergic diseases in children and reached a similar conclusion [21]. Nonetheless, the majority of the studies included in the above-mentioned reviews [20,21] utilised traditional bacterial cultures and polymerase chain reaction (PCR) techniques to characterise the gut microbiota composition and specifically studied the intestinal microbiota–allergy association. A more recent systematic review, in which the authors retrieved studies that utilised genomic sequencing to measure the microbiome composition and diversity, explored the link between the intestinal microbiome and respiratory diseases (including asthma) [22]. The authors highlighted that disruptions in gut microbiota composition alone might not directly lead to respiratory diseases and there is a need for large longitudinal studies [22]. The main objective of the current systematic review was therefore to identify the current evidence for the association between asthma and the upper airway, lower airway and/or gut microbiome in humans and in animals. This study intended to determine the upper airway, lower airway and gut microbiome characteristics commonly associated with asthma. Hence, the findings of this study might have an impact on our understanding of the potential role of the microbiome in asthma development.

## 2. Materials and Methods

We initially performed a non-systematic search within relevant journals for asthma and microbiomes to identify the existing systematic reviews related to these topics. However, the available systematic reviews were generally limited to upper airway or gut microbiome investigations in humans and paid little attention to the lower airway microbiome and animal-based studies. The current review was developed based on the guidelines of the Preferred Reporting Items for Systematic Reviews and Meta-Analyses (PRISMA) [23]. The study team consisted of researchers with experience in microbiology, immunology and respiratory care. It also included a researcher with experience in systematic reviews who was familiar with searching variable databases. The Covidence software from Veritas Health Innovation, Melbourne, Australia (available at https://www.covidence.org/ and accessed on 31 January 2023), was used to manage the retrieved studies, track the status of each study and update the PRISMA flow diagram.

### 2.1. Eligibility Criteria

The eligibility criteria consisted of original articles published in English between inception and February 2022 that addressed asthma diagnosis as an outcome among children up to 18 years old and investigated microbial communities in the upper airway, lower airway or the gut in humans or animals. Studies that addressed asthma diagnosis as a subgroup analysis were also included. The exclusion criteria consisted of studies that examined environmental and/or pollutant microbiomes and asthma or that reported asthma symptoms and/or atopic/allergy diseases without an asthma diagnosis.

### 2.2. Information Sources and Search Strategy

We comprehensively searched the following major electronic databases from 3 to 5 March 2022: PubMed, EBSCO, Science Direct and Web of Science. The search strategy was applied as appropriate for each database. The general search keywords used were: (asthma) AND (microbiome OR dysbiosis OR microbiota). The following filters were applied: age (up to 18 years), language (English) and literature type (original/academic journals). More details on the search strategy are provided in Supplementary File S1.

### 2.3. Selection and Data-Collection Process

All studies were imported to EndNote version X9 and then uploaded to Covidence software. After duplicates were removed, two stages of screening were conducted. First, two independent reviewers screened the titles and abstracts of the imported studies. Second, two independent reviewers conducted full-text screenings for the studies included during the first stage of screening. Finally, independent reviewers performed data extraction based on a data collection form designed specifically to address the objectives of this review (Supplementary Materials Table S1). Conflicts in the screening stages and the data collection process were resolved through regular discussion meetings with all authors.

### 2.4. Data Items

The data collection form (Supplementary Materials Table S1) included the following variables that were extracted from each study: the citation and title of the article, the country where the study was conducted, the study type (human or animal based), the study design, the sample size for each group, the age for each group, the microbiome environment (the upper airway, lower airway and/or the gut), the type of specimen collected for the microbiome analysis, the time of specimen collection (one time point or different time points), the microbiome detection method, the genomic DNA extraction method, the sequencing platform used, the microbial community diversity assessment (α-diversity, β-diversity, or both), the bioinformatics pipeline used and the study findings.

### 2.5. Risk of Bias Assessment

The quality of the included human non-randomised studies was assessed using Newcastle–Ottawa Scale (NOS) tools adapted for each study’s design. Three tools were used: (1) the NOS adapted for cross-sectional studies [24], (2) the NOS for case-control studies and (3) the NOS for cohort studies. The NOS tools were used to assess quality based on different items categorised into three domains (selection, comparability and exposure or outcome). Then, the quality of each study was rated as good, fair or poor by translating the results of the NOS to the Agency for Health Research and Quality standards, as described previously [22]. For animal intervention studies, the Systematic Review Centre for Laboratory Animal Experimentation (SYRCLE) risk of bias tool was used [25]. Details of the tools used are described in Supplementary Materials Table S2.

### 2.6. Synthesis Methods

Due to the nature of the present systematic review, the descriptive data were extracted using a data collection tool that was generated specifically to address the objective of this review (Supplementary Materials Table S1).

## 3. Results

**Figure 1:**
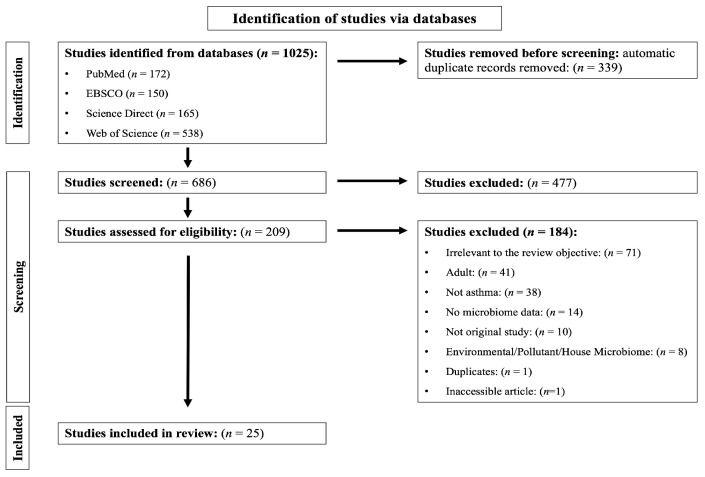
Preferred Reporting Items for Systematic Reviews and Meta-Analyses (PRISMA) flowchart describing the studies excluded and analysed in the current systematic review.

The literature search resulted in a total of 1025 studies, which were uploaded to Covidence. After the duplicates were automatically removed (*n* = 339), 686 studies remained. The titles and abstracts were screened, as a result of which 477 studies were considered irrelevant to the aim of the current review and excluded. The full text of the remaining 209 studies was examined for eligibility. As a result, 184 were excluded for the reasons detailed in Figure 1. The screening phase resulted in 25 studies that met the inclusion criteria and were identified as eligible for inclusion in the present review.

### 3.1. Quality of the Included Studies

Table 1, Table 2 and Table 3 show the quality assessment results of the included human studies (*n* = 22) based on the NOT criteria for case-control, cohort and cross-sectional studies, respectively. Sixteen human studies out of twenty-two were classified as good quality [26,27,28,29,30,31,32,33,34,35,36,37,38,39,40,41], four were classified as fair quality [42,43,44,45] and only two were classified as poor quality [13,46]. The limitations were generally related to the potential selection bias. The quality evaluation for the animal intervention studies (three out of twenty-five) is described in Table 4. The three animal intervention studies [47,48,49] generally indicated the potential performance and detection bias in aspects specifically related to the blinding procedures.

### 3.2. Characteristics of the Included Studies

#### 3.2.1. Clinical Studies

Twenty-two out of the twenty-five studies identified in this review were clinical and examined the upper airway, the lower airway and/or the gut microbiome in healthy controls and/or asthmatic children (Table 5). Ten studies (out of twenty-two) examined the upper airway microbiome [26,29,30,31,33,34,35,36,37,38], while only three studies investigated the lower airway microbiome [13,39,46]. One study analysed both the upper and lower airway microbiomes in healthy controls and children with severe persistent asthma [27]. In a study conducted in 2021, both the upper airway and gut microbiome investigations were performed in healthy controls and asthmatic children [42]. Seven studies (out of twenty-two) analysed faecal specimens to characterise the gut microbiome [28,32,40,41,43,44,45]. The specimen types used to examine the upper airway microbiome were nasal swab [27,29,35,42], nasal wash [33], hypopharyngeal aspirate [30], nasopharyngeal swab [34], nasopharyngeal wash [31], saliva [26] and throat swab [35,36,37,38]. In contrast, the specimen types used to study the lower airway were broncho-alveolar lavage (BAL) [13,27] and sputum [39,46], while faecal specimens were used to study the gut microbiome [28,32,40,41,42,43,44,45].

#### 3.2.2. Animal Intervention Studies

Three out of the twenty-five identified studies were conducted using animal models (Table 6). All three studies used murine models consisting of BALB/c mice [47], Sprague–Dawley (SD) rats [48] and C57BL/6 mice [49]. Regarding asthma induction, for both the BALB/c mouse model [47] and the SD rat model [48], the animals were sensitised by intraperitoneal injections of ovalbumin (OVA) and then challenged by OVA aerosol inhalation. However, there were variations among the methods used in each study, including the frequency and dose schedule of OVA exposure. For the interleukin-13 (IL-13) transgenic (TG) C57BL/6 mouse model, asthma was induced by lung-specific IL-13 overexpression [49]. The first animal intervention study performed 16S rRNA sequencing on both the nasal lavage fluid and BAL to characterise the upper and lower airway microbiomes in mice with OVA-induced asthma [47]. The second study extracted the lung tissues from rats with allergic asthma to characterise the lower airway microbiome [48]. BAL, lung tissue and faecal specimens were collected from IL-13 transgenic mice simulating chronic asthma to examine both the lower airway and gut microbiomes [49].

**Table 5:** Overview of the included clinical studies that investigated microbiome and asthma.

Citation and Title of the Article	Country	Study Design	Sample Size	Age	Sample Collected	Time of Sample Collection	Microbiome Detection Method	Genomic DNA Extraction Method	Sequencing Platform	Microbiome Diversity Assessment	Bioinformatics Pipeline Used	Findings
Upper airway microbiome												
[29] ‘Longitudinal Changes in Early Nasal Microbiota and the Risk of Childhood Asthma’	Finland	Cohort	2-month visit: *n* = 704 13-month visit: *n* = 665 24-month visit: *n* = 570	2-month visit: 2.5 (2.4–2.7) 13-month visit: 13.5 (13.1–13.9) 24-month visit: 25.0 (24.6–25.5)	Nasal swabs	3 time points: 2, 13 and 24 months	16S rRNA gene sequencing (V4 region)	Automated MagNA Pure 96 System	Illumina MiSeq	α-diversity: Shannon index and β-diversity: Bray–Curtis	UPARSE OUT clustering	Insignificant difference in α-diversity as well as β-diversity between children who developed asthma by age 7 years and those who did not. ↑ Relative abundance of *Haemophilus* over age 2 to 13 months was associated significantly with higher risk of asthma. ↑ Relative abundance of *Lactobacillus* at age 2 months was associated significantly with lower risk of asthma.
[30] ‘Infant airway microbiota and topical immune perturbations in the origins of childhood asthma’	Denmark	Cohort	700	The cohort was followed up from the age of 1 week until 6 years of life	Hypopharyngeal aspirates	Different time-points: Hypopharyngeal aspirates were obtained at ages 1 week, 1 month and 3 months	16S rRNA gene sequencing (V4 region)	PowerMag Soil DNA Isolation Kit	Illumina MiSeq	α-diversity: Shannon index and β-diversity: Bray–Curtis and UniFrac, weighted	Mothur	At age 1 month: ↑ α-diversity and a difference in β-diversity in children who developed asthma in the first 6 years of life compared to those who did not. ↑ Relative abundance of *Veillonella* and *Prevotella* at age 1 month were associated significantly with asthma development by age 6 years. At ages 1 week and 3 months: Insignificant association between α- or β-diversity or any taxa and the development of asthma.
[33] ‘Pediatric asthma comprises different phenotypic clusters with unique nasal microbiotas’	USA	Cross-section	163 children and adolescents	Age for all participants years (SE): 11.0 (0.3)	Nasal washes	205 nasal washes. 1 time point: 163 sample 2 time points: 42 samples (patients came back for an additional visit (5.5 to 6.5 months apart), and one additional sample was taken)	16S rRNA gene sequencing (V4 region)	QIAGEN QIAamp DNA Kit	Illumina MiSeq	α-diversity: Shannon index, ACE indices, and Faith’s phylogenetic diversity index and β-diversity: UniFrac (unweighted and weighted), Bray-Curtis, and Jaccard distances	Mothur	Operational taxonomic units of pathogenic *Moraxella*, *Staphylococcus*, *Streptococcus* and *Haemophilus* were present in 95% of nasal microbiotas in asthmatics.
[31] ‘Nasopharyngeal Microbiome Diversity Changes over Time in Children with Asthma’	USA	Cohort	40 children and adolescents	6–18 years; mean = 11 years	Nasopharyngeal washes	Two samples (5.5 to 6.5 months apart)	16S rRNA gene sequencing (V4 region)	QIAGEN QIAamp DNA Kit	Illumina MiSeq	α-diversity: Good’s coverage, Chao1, Shannon indices, and Faith’s phylogenetic diversity index and β-diversity: UniFrac (unweighted and weighted)	Mothur	The nasopharyngeal core microbiome of asthmatics at the 95% level: *Moraxella*, *Staphylococcus*, *Streptococcus*, *Haemophilus*, *Fusobacterium*. 86% of the total reads in asthmatics were: *Moraxella*, *Staphylococcus*, *Dolosigranulum*, *Corynebacterium*, *Prevotella*, *Streptococcus*, *Haemophilus*, *Fusobacterium* and a Neisseriaceae.
[34] ‘Different functional genes of upper airway microbiome associated with natural course of childhood asthma’	Korea	Cross-section	Healthy children (controls), *n* = 31 Children with asthma, *n* = 30 Children with asthma in remission, *n* = 30	Years Healthy children (controls): 7.1 ± 1.1 Children with asthma: 8 ± 0.9 Children with asthma in remission: 7.6 ± 1.4	Nasopharyngeal swabs	1 time point	16S rRNA gene sequencing (V1-V3 region)	PowerMag Microbiome RNA/DNA isolation kit (MP Biomedicals, Santa Ana, CA, USA)	Illumina TruSeq DNA	α-diversity: Shannon index and β-diversity: UniFrac (unweighted and weighted)	No mention	Control group: ↑ Relative abundance of *Haemophilus* and *Moraxella.* Asthma and remission groups: ↑ Relative abundance of *Streptococcus*, *Dolosigranulum*, and *Corynebacterium.* Asthma group: ↑ Relative abundance of *Staphylococcus.*
[26] ‘Bacterial salivary microbiome associates with asthma among African American children and young adults’	USA	Case control	Asthma cases, *n* = 57 Healthy controls, *n* = 57	Asthma case: 15.6 ± 3.3 Healthy controls: 15 ± 3.9	Saliva	1 time point	16S rRNA gene sequencing (V4 region)	Oragene DNA Discover OGR-500 self-collection kits	Illumina MiSeq	α-diversity: Shannon index	QIIME	Significant difference in α-diversity between asthma cases and healthy controls. Asthma cases: ↓ Relative abundance of *Streptococcus.* ↑ Relative abundance of *Veillonella.* Healthy controls: ↑ Relative abundance of *Streptococcus.* ↓ Relative abundance of *Veillonella.*
[35] ‘Bacterial microbiota of the upper respiratory tract and childhood asthma’	Europe	Cross-section	Throat swabs: Children with asthma, *n* = 125 Controls, *n* = 202 Nasal swabs: Children with asthma, *n* = 39 Controls, *n* = 29	6 to 12 years	Nasal and throat swabs	1 time point	16S rRNA gene sequencing (V3-V5 region)	QIAmp DNA Mini Kit	Pyrosequencing, Roche 454-GS FLX Titanium	α-diversity: Shannon index and β-diversity: Unweighted UniFrac distances	QIIME	Asthma was associated with alterations in nasal (not throat) microbiome. Asthmatic children versus controls: ↓ α- and β-diversity and lower abundance of *Moraxella* of nasal microbiome.
[36] ‘Integration of metagenomics-metabolomics reveals specific signatures and functions of airway microbiota in mite-sensitized childhood asthma’	China	Cross-section	Control: *n* = 28 Asthma: *n* = 27	Years Control: 4.54 ± 0.3 Asthma: 4.32 ± 0.85	Throat swabs	1 time point. Asthma case: swabs were collected before inhaled or nasal administration of corticosteroids for regular daily treatment. Control: no mention.	Shotgun metagenome sequencing	FastDNA SPIN Kit for Soil (MP Biomedical)	Illumina HiSeq	α-diversity: Shannon index and β-diversity: Bray–Curtis index	Metagenome assembly by MEGAHIT and contig binning by MetaBAT	No difference in α-diversity between asthma and control groups, but β-diversity difference was detected between the two groups. Asthma group: Predominance of *Neisseria elongate.* Control group: Significant enrichment of *Eubacterium sulci*, *Leptotrichia wadei* and *Prevotella* spp.
[37] ‘Integrated metabolic and microbial analysis reveals host-microbial interactions in IgE-mediated childhood asthma’	Taiwan	Cross-section	Asthma (non-atopic, lowly sensitize): *n* = 15 Asthma (non-atopic, highly sensitize): *n* = 13 Healthy controls: *n* = 25	Years Asthma (non-atopic, lowly sensitized): 3.7 ± 0.6 Asthma (non-atopic, highly sensitized): 3.5 ± 0.7 Healthy controls: 3.6 ± 0.7	Throat swabs	1 time point, no time specified	16S rRNA gene sequencing (V3-V4 region)	FastDNA Spin Kit for Soil (MP Biomedical, Solon, OH, USA)	Illumina HiSeq 2500	α-diversity: Shannon index and Chao1 index	QIIME	No statistically significant difference in airway taxa composition between asthma and healthy controls. Highly sensitized asthma children: ↓ Relative abundance of *Dialister*, *Streptococcus*, *Prevotella*, *Tannerella*, *Atopobium* and *Ralstonia.*
[38] ‘Comparison of Oropharyngeal Microbiota from Children with Asthma and Cystic Fibrosis’	Germany	Cross-sectional	Control children: *n* = 62 Children with asthma: *n* = 27 Children with cystic fibrosis (CF): *n* = 57	Years (min–max) Control: 10.1 (8–12) Asthma: 10 (8–12) CF: 10.61 (6–12)	Throat swabs	1 time point	16S rRNA gene sequencing (V4 region)	QIAamp Mini Kit	Illumina MiSeq system	α-diversity: Shannon index and Chao1 index and β-diversity: Morisita–Horn similarity index	Mothur	High level of similarity was detected between control, asthma and CF groups. Core microbiome in healthy controls, children with asthma and CF: *Prevotella*, *Streptococcus*, *Neisseria*, *Veillonella* and *Haemophilus.*
Lower airway microbiome												
[13] ‘Disordered microbial communities in asthmatic airways’	Ireland	Cross-sectional	Difficult asthma, *n* = 13 Non-asthmatic controls, *n* = 7	Asthmatic children: 11.8 ± 2.8 years Controls: 11.3 ± 5.7 years	Bronchoalveolar lavage (BAL)	1 time point, time not specified	16S rRNA gene sequencing (V3 region) and cloning	DNeasyn (Qiagen)	No mention	α-diversity: Chao1 index	DOTUR program	Asthmatic children: Significant increase in Proteobacteria Children with difficult asthma: ↑ *Staphylococcus* spp. Controls: ↑ Bacteroidetes (*Prevotella* spp.).
[46] ‘Altered respiratory microbiota composition and functionality associated with asthma early in life’	United Arab Emirates	Case control	Paediatric asthmatic: *n* = 11 Paediatric healthy: *n* = 9	Years, mean (SD, range) Paediatric asthmatic: 6.7 (4.1, 12) Paediatric healthy: 8 (3.1, 8)	Sputum	1 time point: Spontaneous coughed up sputum (expectorated phlegm/mucous) was the first preference of sample collection whenever possible in all subjects.	16S rRNA gene sequencing for bacteria (V4 region) ITS2-gene based microbial profiling for fungi	MoBio PowerMag Soil DNA Isolation	Illumina MiSeq	α-diversity: Shannon index and β-diversity: Bray-Curtis index	Mothur	Asthmatic versus non-asthmatic controls: Significant difference of bacteria and fungi between the two groups. Significant difference in Bacteroidetes, Firmicutes, Fusobacteria and Proteobacteria. Paediatric asthma: ↑ Relative abundance in *Streptococcus* spp. and *Moraxella* spp. Difference in Ascomycota, Basidiomycota phyla and other unclassified fungi. ↓ *Penicillium aethiopicum* and *Alternaria* spp.
[39] ‘Gram-negative microbiota is related to acute exacerbation in children with asthma’	Korea	Cross-section	Total children, *n* = 95 Children with asthma exacerbation: *n* = 22 Children with stable asthma: *n* = 67 Controls: *n* = 6	Years Asthma exacerbation: 9.0 (6.4/10.9) Stable asthma: 8.0 (6.6/9.7) Controls: 13.2 (10.7/14.9)	Sputum	1 time point	16S rRNA gene sequencing (V3-V4 region)	FastDNA SPIN Kit for Soil (MP Biomedicals, USA)	Illumina MiSeq	α-diversity: ACE, Chao1, Jackknife, NPShannon, Shannon and Simpson and β-diversity: Jensen–Shannon, Bray–Curtis, Generalised UniFrac, and UniFrac indices	No mention	No difference in α-diversity detected between asthma exacerbation and stable asthma children. Significant difference in β-diversity detected between asthma exacerbation and stable asthma children. Asthma exacerbation: Phylum level: ↑ Abundance of Proteobacteria. ↓ Abundance of Saccharibacteria and Actinobacteria. Genus level: ↑ Abundance of *Veillonella*, *Neisseria*, *Haemophilus*, *Fusobacterium*, *Oribacterium*, *Campylobacter* and *Capnocytophaga* ↓ *Saccharimonas*, *Rothia*, *Porphyromonas*, *Gemella* and *Actinomyces.*
Upper and lower airway microbiome												
[27] ‘Integrative study of the upper and lower airway microbiome and transcriptome in asthma’	USA	Case control	Children with severe persistent asthma: *n* = 27 Healthy controls: *n* = 27	Years Children with severe persistent asthma: 11, IQR 8 Healthy controls: 13, IQR 6	Nasal swabs BAL	1 time point	16S rRNA gene sequencing (V3-V4 region)	Qiagen DNeasy Mini Kit	Illumina MiSeq	α-diversity: Shannon index and β-diversity: UniFrac distance index	QIIME	α-diversity was higher in bronchial (BAL) versus nasal. Significant difference in β-diversity detected between bronchial (BAL) and nasal. Asthmatic children Nasal microbiome: *Moraxella* and *Alloiococcus* are hub genera. Bronchial microbiome: no hubs. Nasal *Streptococcus* was enriched in children with persistent asthma versus healthy controls.
Upper airway and gut microbiome												
[42] ‘Altered IgA Response to Gut Bacteria Is Associated with Childhood Asthma in Peru’	Peru	Case control	Asthma: *n* = 40 Control children: *n* = 40	Years Asthma: 14.6 ± 1.5 Controls: 13.3 ± 2.3	Nasal swabs and faecal specimens	1 time point: Biospecimens samples (nasal swabs and faecal) were collected the same day of the home visit or during the same week	16S rRNA gene sequencing (V4 region)	Faecal specimens ethanol-based method Nasal swabs: no information was provided	Illumina MiSeq	α-diversity: Shannon index and Renyi entropy and β-diversity: Bray–Curtis and UniFrac distances	DADA 2	α-and β-diversity of faecal as well as nasal swabs showed no difference between asthma and controls.
Gut microbiome												
[32] ‘Reduced diversity of the intestinal microbiota during infancy is associated with increased risk of allergic disease at school age’	Denmark	Cohort	411 infants	Full-term infants born at >36 week and were followed until 6 years	Faecal specimens	2 time points: At 1 month old and 12 months old	16S rRNA gene and denaturing gradient gel electrophoresis (V3 region)	QIAamp DNA stool Mini Kit (Qiagen, Hilden, Germany)	None	Band richness and principal component analysis	BioNumerics software 4.50	No association between bacterial diversity of the infant’s gut microbiota and asthma in the first 6 years of life.
[28] ‘Shifts in Lachnospira and Clostridium sp. in the 3-month stool microbiome are associated with preschool age asthma’	Canada	Case control	Total 76. Asthmatic: *n* = 39 Healthy control: *n* = 37	Follow up from birth till 4 years.	Faecal specimens	2 time points: 3 months and 1 year of age	16S rRNA gene sequencing (V3 region)	Mo Bio dry bead tubes (Mo Bio Laboratories)	Illumina Hi-Seq. 2000	α-diversity: Shannon index and β-diversity	Mothur	At 3 months asthmatic children: ↓ Abundance of *Lachnospira.* ↑ Abundance of *Clostridium neonatale.* Negative association between the ratio of *Lachnospira* and *Clostridium neonatale* and asthma risk by 4 years of age.
[43] ‘Early infancy microbial and metabolic alterations affect risk of childhood asthma’	Canada	Longitudinal nested case control	Control: *n* = 74 Atopy and wheeze: *n* = 22 Atopy only: *n* = 87 Wheeze only: *n* = 136	Baseline: 1 year of age Follow-up: 3 years of age	Faecal specimens	2 time points: at 3 months and 1 year	16S rRNA gene sequencing (V3 region)	Qiagen DNA Stool Mini Kit	Illumina HiSeq 2000	α-diversity: Shannon index	Mothur	No significant difference in α-diversity among four groups. Children at risk of asthma: ↓ Relative abundance of *Lachnospira*, *Veillonella*, *Faecalibacterium* and *Rothia.*
[44] ‘Gut microbial-derived butyrate is inversely associated with IgE responses to allergens in childhood asthma’	Taiwan	Case control	Children with rhinitis: *n* = 27 Children with asthma: *n* = 34 Healthy controls, *n* = 24	Years Controls: 5.7 ± 0.8 Rhinitis: 6.0 ± 0.9 Asthma: 5.6 ± 0.9	Faecal specimens	1 time point. Time not specified.	16S rRNA gene sequencing (V3-V4 region)	FastDNA Spin Kit for Faeces (MP Biomedical)	Illumina HiSeq 2500	α-diversity: species richness	QIIME	Children with rhinitis and asthma versus healthy controls: ↓ Relative abundance of Firmicutes. ↓ Relative abundance of *Faecalibacterium*, *Roseburia*, *SMB53* and *Dialister.* ↑ Relative abundance of *Escherichia*, *Enterococcus* and *Clostridium.*
[40] ‘Gut microbial dysbiosis is associated with allergen-specific IgE responses in young children with airway allergies’	Taiwan	Cross-section	Controls: *n* = 26 Asthma: *n* = 35 Rhinitis: *n* = 28	Controls: 5.6 ± 0.8 Asthma: 5.5 ± 0.9 Rhinitis 5.9 ± 0.9	Faecal specimens	1 time point	16S rRNA gene sequencing (V3-V4 region)	FastDNA Spin Kit for Faeces (MP Biomedical, Solon, OH, USA)	Illumina HiSeq 2500	α-diversity: Shannon index and Chao 1 index and β-diversity: Bray–Curtis and Weighted UniFrac distance	QIIME	Relatively lower α-diversity in allergic disease than control (insignificant). No significant difference in β-diversity in allergic airway disease. Children with asthma and allergic rhinitis versus healthy controls: ↓ Relative abundance of Firmicutes. ↓ Relative abundance of *Dorea* spp. ↑ Relative abundance of *Clostridium* spp.
[45] ‘Neonatal gut microbiota associates with childhood multisensitized atopy and T cell differentiation’	USA	Cohort	1 month: *n* = 130 infants 6 months: *n* = 168 infants	1 month and 6 month infants	Faecal specimens	2 time points: 1 month and 6 months.	16S rRNA gene sequencing (V4 region) (ITS)2 rRNA sequencing for fungi	In-house kit: Modified cetyltrimethylammonium bromide buffer-based protocol	Illumina MiSeq	α-diversity: Shannon index and β-diversity: Unweighted UniFrac distance and Bray–Curtis	QIIME	The highest risk group: ↓ Relative abundance of *Bifidobacterium*, *Akkermansia* and *Faecalibacterium.* ↑ Relative abundance of *Candida* and *Rhodotorula.*
[41] ‘Correlations of Inflammatory Factors with Intestinal Flora and Gastrointestinal Incommensurate Symptoms in Children with Asthma’	China	Cross-section	Asthmatic group: *n* = 70 Control group: *n* = 25	Years Asthmatic group: 9.03 ± 2.01 Control group: 8.12 ± 2.13	Faecal specimens	1 time point (exact time was not mentioned)	SYBR GREEN I fluorescence quantitative polymerase chain reaction	No mention	Not applicable	Total load of bacteria between observation group and control group	Not applicable	The total load of bacteria: asthmatic group > control group Asthmatic group: ↓ Load of *Bifidobacterium* and *Lactobacillus.* ↑ Load of *Escherichia coli*, *Helicobacter pylori*, *Streptococcus* and *Staphylococcus.* Control group: ↑ Load of *Bifidobacterium* and *Lactobacillus*. ↓ Load of *Escherichia coli*, *Helicobacter pylori*, *Streptococcus* and *Staphylococcus.*

### 3.3. Microbiome Quantification

The 25 identified studies in the current review analysed the bacteriome (Table 5). Two studies investigated only the mycobiome in addition to the bacteriome [45,46]. However, none of the identified studies evaluated the virome. Of the 25 included studies, 23 (92.0%) utilised 16S rRNA gene sequencing to characterise bacterial communities in the upper airway, lower airway or faecal specimens, as shown in Table 5 and Table 6. These studies targeted different sequencing regions on 16S rRNA, consisting of region V3 (*n* = 3; 13.0%) [28,43], V4 (*n* = 9; 39.1%) [26,29,30,31,33,35,38,42,45,46], V1–V3 (*n* = 1; 4.3%) [34], V3–V4 (*n* = 6; 26.0%) [27,37,39,40,47], V4–V5 (*n* = 1; 4.3%) [48] and V3–V5 (*n* = 1; 4.3%) [35]. One study did not indicate the targeted sequencing region [49]. Additionally, a single study used 16S rRNA gene sequencing with cloning [13] and another study used the 16S rRNA gene and denaturing gradient gel electrophoresis [32]. In both studies, V3 was the targeted sequencing region [13,32]. In addition, one study used shotgun metagenome sequencing [36] and another study used the SYBR GREEN I fluorescence quantitative polymerase chain reaction method [41] to characterise the microbiome. In the two studies that characterised the mycobiome, the internal transcribed spacer region (ITS)2 of the rRNA gene was amplified and sequenced using the Illumina MiSeq platform (Illumina, Inc., San Diego, CA, USA) [45,46].

### 3.4. Diversity Assessments

As shown in Table 5 and Table 6, 18 out of the 25 identified studies (72.0%) assessed both the α- and β-diversity of the upper airway, lower airway and/or gut microbiome. These studies have reported contradictory findings related to α- and β-diversity. For instance, an insignificant difference was observed in both α- and β-diversity between asthmatic children and non-asthmatics [29,40,42]. On the contrary, a significant difference in α- and β-diversity of the upper airway, lower airway and/or gut microbiome was detected between asthmatic children and non-asthmatics [27,30,35,46,48]. Five studies evaluated only the α-diversity of the microbiome in the upper airway, lower airway and/or gut microbiome (20.0%) [13,26,37,43,44] and demonstrated conflicting data. For example, Espuela-Ortiz and colleagues (2019) reported a significant difference in the α-diversity of the upper airway microbiome between asthma cases and healthy controls [26]. Another study detected insignificant differences in the airway taxa composition between asthma patients and healthy controls [37]. However, Bisgaard and colleagues (2011) estimated band richness and conducted principal component analysis (PCA), which resulted in no association between the bacterial diversity of the infant’s gut microbiome and asthma development in the first 6 years of life [32]. The total load of bacteria for asthmatic children and healthy controls was calculated, and the authors reported a higher bacterial load in asthmatic children than in the healthy control group [41].

### 3.5. Microbiome Outcome 

#### 3.5.1. Human Studies

The data presented in Table 5 indicates that the microbiome in the upper airways of asthmatic children has a significantly high relative abundance of *Moraxella*, *Staphylococcus*, *Streptococcus*, *Haemophilus*, *Fusobacterium*, *Dolosigranulum*, *Corynebacterium*, *Veillonella* and *Neisseria elongate* [26,31,33,34,36]. However, a significantly low relative abundance of *Streptococcus*, *Moraxella*, *Dialister*, *Prevotella*, *Tannerella*, *Atopobium* and *Ralstonia* was identified in the upper airways of asthmatic children [26,35,37]. An increased relative abundance of *Haemophiles* in children aged 2 to 13 months was significantly associated with a higher risk of asthma development [29]. An additional study reported that a high relative abundance of *Veillonella* and *Prevotella* at age 1 month was significantly associated with asthma development by age 6 [30]. However, a significantly high abundance of *Lactobacillus* at age 2 months was associated with a lower risk of asthma development, suggesting that this bacterium plays a protective role [29].

The lower airway microbiome indicated a significant increase in Protobacteria in asthmatic children, particularly in asthma exacerbation cases [13,39], while a significant decrease in Saccharibacteria and Actinobacteria was detected [39]. Moreover, asthma exacerbation was associated with a high relative abundance of *Veillonella*, *Neisseria*, *Haemophilus*, *Fusobacterium*, *Oribacterium*, *Campylobacter* and *Capnocytophaga* in sputum [39]. However, *Saccharimonas*, *Rothia*, *Porphyromonas*, *Gemella* and *Actinomyces* were detected with low significant relative abundance in asthma exacerbation cases [39]. A high relative abundance of *Streptococcus*, *Moraxella* and *Staphylococcus* was identified in asthmatic children, with the latter detected in difficult asthma cases [13,46]. A mycobiome analysis revealed a significantly low abundance of *Penicillium aethiopicum* and *Alternaria* spp. in sputum specimens collected from asthmatic children [46].

The gut microbiome studies that examined the faecal specimens of asthmatic children revealed a significant increase in the relative abundance of *Clostridium*, *Escherichia* and *Enterococcus* [32,40,44]. In addition, a higher load of *E. coli*, *Helicobacter pylori*, *Streptococcus* and *Staphylococcus* was detected in the faecal specimens of asthmatic children [41]. A lower load of *Bifidobacterium* and *Lactobacillus* was detected in the faecal specimens of the same group, indicating that these bacteria play a protective role [41]. The mycobiome analysis of faecal specimens obtained from infants revealed a high relative abundance of *Candida* and *Rhodotorula*, which were associated with a high risk of developing asthma [45]. In contrast, the relative abundance of *Lachnospira*, *Faecalibacterium*, *Roseburia*, *SMB53*, *Dialister* and *Dorea* was significantly decreased in asthmatic children [28,40,44]. *Lachnospira*, *Veillonella*, *Faecalibacterium*, *Rothia*, *Bifidobacterium* and *Akkermansia* were significantly decreased in high-risk children [43,45].

#### 3.5.2. Animal Intervention Studies

A respiratory microbiome analysis identified an increase in the relative abundance of *Pseudomonas* spp. during the acute inflammatory stage, while *Staphylococcus* spp. and *Cupriavidus* spp. increased during the airway remodelling stage in mice with OVA-induced asthma [47]. The bacterial phylum Firmicutes were detected at higher levels in the lower airway (lung tissues) microbiomes of rats with allergic asthma [48]. Proteobacteria and Cyanobacteria phyla were identified at higher levels in the lungs of IL-13 TG mice [49]. The microbiome analysis of faecal specimens extracted from IL-13 TG mice reflected a lower level of Firmicutes and Protobacteria, whereas the lung microbiome indicated a low level of Bacteroidetes [49]. 

## 4. Discussion

The aims of the current study were to examine the association between asthma and the upper airway, lower airway and/or gut microbiome in humans and animals and identify the characteristics of the upper airway, lower airway and the gut microbiome commonly associated with asthma.

The data presented in this review demonstrated that the clinical specimens collected from both the control and asthmatic children were mostly from the upper airway (i.e., a nasal swab, nasal wash, hypopharyngeal aspirate, nasopharyngeal swab, nasopharyngeal wash, throat swab and saliva). Only three studies collected specimens from the lower airway (BAL and sputum) [13,39,46], and one contained specimens from both the upper and lower airways [27]. The limited number of lower airway microbiome studies might contribute to the difficulty in collecting lower airway human specimens (specifically from healthy children) as it is more convenient to collect specimens from the upper airway.

Evidence of the association between asthma and changes in the upper and lower airways and/or gut microbiome was synthesized. The phyla Proteobacteria (*Haemophilus*, *Moraxella*, *Neisseria*, *Campylobacter*, *Escherichia* and *Helicobacter*) and Firmicutes (*Veillonella*, *Staphylococcus*, *Streptococcus*, *Dolosigranulum*, *Oribacterium*, *Alloiococcus*, *Clostridium* and *Enterococcus*) were identified as being significantly higher in the asthmatic children [13,39] compared with the healthy controls. These findings confirm the previous observations that Proteobacteria (*Haemophilus*, *Moraxella* and *Neisseria*) and Firmicutes (*Staphylococcus* and *Streptococcus*) were the most abundant bacteria in asthmatic children [50].

A previous literature review performed in 2019 reported that the most dominant genera in the upper airways of infants are *Corynebacterium*, *Dolosigranulum*, *Haemophilus*, *Moraxella*, *Staphylococcus* and *Streptococcus* [51]. However, in this study, we found that the upper airway microbiome in 1-month-old infants indicated an increase in the relative abundance of *Veillonella* and *Prevotella*, which were associated with asthma development later in life [30]. Both genera were considered normal flora of the upper respiratory system and their increased abundance in infants suggests their potential involvement in asthma development later in life [30]. Furthermore, the upper airway microbiome in infants ranging in age between 2 and 13 months indicated a higher abundance of *Haemophilus*, which was associated with a higher risk of asthma development later in life [29]. This substantiates the results of a previous review, which highlighted that dysregulated *Haemophilus* was common in asthmatic children [52].

As shown in Table 5, the upper airways of asthmatic children have a significant high relative abundance of *Moraxella*, *Staphylococcus*, *Streptococcus*, *Haemophilus*, *Fusobacterium*, *Dolosigranulum*, *Corynebacterium*, *Veillonella* and *Neisseria elongate* and a high relative abundance of *Streptococcus*, *Moraxella* and *Staphylococcus* was determined in their lower airways. The above-mentioned bacteria are known as normal human microbiota in the respiratory tract [53]. Furthermore, *Staphylococcus*, *Streptococcus* and *Haemophilus*, followed by *Moraxella* and *Veillonella* were the most frequently reported bacterial genera in the respiratory system of asthmatic children (Table 5). Previous studies have highlighted that the clinical characteristics of asthma patients and the type of immune response stimulated by aeroallergens influence airway microbiome composition [54]. For instance, *Moraxella catarrhalis*, a species of *Haemophilus*, and *Streptococcus* were the predominant respiratory tract bacteria in patients with severe asthma and corticosteroid resistance [54]. The literature points to a lack of metabolomic investigations of the association between the metabolic characteristics of these dysbiotic bacteria and asthma phenotypes and treatment prognosis. For instance, asthma patients with steroid resistance might have a higher abundance of airway microbial communities that can degrade steroids [55].

It has been established in the literature that the dysbiosis of the normal gut microbiome plays an important role in the development of immune disorders, including asthma [56,57]. This is explained by the key role of the gut microbiome in shaping the human immune system [55]. Differences in the gut microbiome in terms of composition and diversity were previously reported between healthy and asthmatic children [52]. In this study, the high relative abundance of the genus *Clostridium* was detected in faecal specimens collected from asthmatic children in three studies [28,40,44]. Previous studies have shown that the *Clostridium species* have an impact on the host’s immune system [7]. In addition, infant colonization with *Clostridium species* is associated with a higher risk of allergy development [7]. This substantiates the findings of the current review as a predominance of the *Clostridium species* was detected during early childhood and was associated with asthma development [28].

The studies analysed in this review lacked consistency in reporting their findings. Some of the studies on bacterial communities in airways and/or gut have identified most of the detected bacterial taxa at the phylum level [13,39,40,44,46], whereas the others have identified the detected taxa at the genus level [26,27,28,29,30,31,33,34,35,36,37,38,41,43,45] (Table 5). Due to this inconsistency, making an accurate comparison of these studies became challenging. Moreover, bacteria belonging to different genera under the same phylum might have different effects on a host. For instance, this review revealed that the genus *Lactobacillus*, which belongs to the phylum Firmicutes, is associated with a low risk of asthma development, suggesting that the bacteria under this phylum play a protective role in asthma. By contrast, other genera under the same phylum Firmicutes, such as *Veillonella*, are significantly associated with asthma development later in life, suggesting their contributory role in asthma development. Therefore, it has been recommended that the use of reporting guidelines (i.e., the Strengthening the Organization and Reporting of Microbiome Studies [STORMS] checklist) must be adopted in future human microbiome studies [58].

Contradictory findings on microbiome diversity were reported by the included clinical studies (Table 5). Of the 22 clinical studies, 15 determined both the α- and β-diversity of the upper airway, lower airway and/or gut microbiomes, but they reported conflicting findings on α- and β-diversity between the asthmatic and non-asthmatic children. As depicted in Table 5, the clinical studies were conducted in different geographic locations, including North America, Europe, Asia, and Middle East, and they analysed clinical specimens obtained from different ethnic groups. The literature highlighted that the gut microbiome composition is associated with ethnicity and geography [59]. Furthermore, the sample sizes in 16 clinical studies were heterogeneous, with minimum and maximum sample sizes of 20 [46] and 923 [29] children, respectively. This sample size variation might have contributed to the variations in the diversity metrics [60]. The clinical studies also varied with respect to technical protocols, next-generation sequencing platforms and bioinformatics pipelines, as described in Table 5, and these variations might have influenced the quality of the obtained microbiome data [61].

There is limited literature on the use of animal intervention studies to examine the association between asthma development and microbiomes. The criteria related to random housing, blinding and random outcome assessment may hinder the research on such studies as the validity might be compromised. The quality assessment of animal intervention studies included in this review [47,48,49] generally indicated the potential performance and detection bias in aspects related to the blinding procedures, which might influence the validity of the results of these studies [47,48,49]. Furthermore, the current review indicated a lack of microbiome data related to viruses, archaea and micro-eukaryotes (such as protozoa). The characterization of these rare microbiome components might have a valuable impact on our understanding of asthma development.

## 5. Conclusions

The phyla Proteobacteria and Firmicutes were identified as being significantly higher in the asthmatic children compared with the healthy controls. The high relative abundance of *Veillonella*, *Prevotella* and *Haemophilus* in the microbiome of the upper airway in early infancy was associated with a higher risk of asthma development later in life. Gut microbiome analyses indicated that a high relative abundance of the genus *Clostridium* in early childhood might be associated with asthma development later in life. The findings reported here serve as potential microbiome signatures associated with an increased risk of asthma development. There is a need for human studies targeting the lower airway as well as well-designed animal intervention studies to further identify high-risk infants, which will help in design strategies and prevention mechanisms to avoid asthma early in life.

## Figures and Tables

**Table 1:** Newcastle–Ottawa Scale for the human case-control studies.

Citation	Selection				Comparability	Exposure			Total	Rate
	Is the Case (Asthma) Definition Adequate?	Representativeness of the Cases	Selection of Controls	Definition of Controls	Comparability of Cases and Controls on the Basis of the Design or Analysis	Ascertainment of Exposure	Same Method of Ascertainment for Cases and Controls	Non-Response Rate		
[26]	1	1	1	1	1	1	1	1	8	Good
[46]	1	0	0	0	1	1	0	0	3	Poor
[27]	1	1	1	1	1	1	1	1	8	Good
[42]	1	1	0	1	1	1	1	0	6	Fair
[28]	1	1	1	1	2	1	1	0	8	Good
[43]	1	1	0	0	2	1	1	0	6	Fair
[44]	1	0	0	1	1	1	1	1	6	Fair

**Table 2:** Newcastle–Ottawa Scale for the human cohort studies.

Citation	Selection				Comparability	Outcome			Total	Rate
	Representativeness of the Exposed Cohort	Selection of the Non-Exposed Cohort	Ascertainment of Exposure to Implants	Demonstration That Outcome of Interest (Asthma) Was Not Present at Start of Study	Comparability of Cohorts on the Basis of the Design or Analysis	Assessment of Outcome	Was Follow Up Long Enough for Outcome to Occur	Adequacy of Follow-Up of Cohorts		
[29]	1	0	1	1	2	1	1	1	8	Good
[30]	1	1	1	1	2	1	1	1	9	Good
[31]	1	1	1	1	1	1	1	1	8	Good
[32]	1	1	1	1	1	1	1	1	8	Good
[45]	1	1	0	1	1	0	1	1	6	Fair

**Table 3:** Newcastle–Ottawa Scale for human cross-sectional studies.

Citation	Selection				Comparability	Outcome		Total	Rate
	Representativeness of the Sample	Sample Size	Non-Respondents	Ascertainment of the Exposure	The Subjects in Different Outcome Groups Are Comparable, Based on the Study Design or Analysis. Confounding Factors Are Controlled	Assessment of Outcome	Statistical Test		
[33]	1	1	0	1	2	2	1	8	Good
[34]	1	1	0	1	2	2	1	8	Good
[35]	1	0	1	2	2	1	1	8	Good
[36]	1	0	1	2	1	1	1	7	Good
[37]	1	0	1	2	2	2	1	9	Good
[38]	1	1	0	1	2	2	1	8	Good
[13]	0	0	0	0	0	0	1	1	Poor
[39]	1	1	0	1	2	2	1	8	Good
[40]	1	0	1	2	1	1	1	7	Good
[41]	1	0	1	2	0	2	1	7	Good

**Table 4:** The systematic review centre for the laboratory animal experimentation risk of the bias assessment tool for animal studies.

Citation	Selection Bias			Performance Bias		Detection Bias		Attrition Bias	Reporting Bias	Other
	Sequence Generation	Baseline Characteristics	Allocation Concealment	Random Housing	Blinding	Random Outcome Assessment	Blinding	Incomplete Outcome Data	Selective Outcome Reporting	Was the Study Apparently Free of Other Problems That Could Result in High Risk of Bias?
[47]	Yes	Yes	Unclear	Yes	Unclear	Unclear	Unclear	No	No	No
[48]	Yes	Yes	Yes	Yes	Unclear	Yes	Unclear	No	No	No
[49]	Unclear	Yes	Unclear	Unclear	Unclear	Unclear	Unclear	No	No	Unclear

**Table 6:** Overview of the included animal-based studies that investigated the microbiome and asthma.

Citation and Title of the Article	Country	Sample Size	Age	Type of Sample Collected	Time of Sample Collection	Microbiome Detection Method	Genomic DNA Extraction Kit	Sequencing Platform	Microbiome Diversity Assessment	Bioinformatics Pipeline Used	Study Findings
Upper and lower airway microbiome											
[47] ‘Respiratory Microbiota Profiles Associated with the Progression from Airway Inflammation to Remodelling in Mice With OVA-Induced Asthma’.	China	Female BALB/c mice: *n* = 30 Control group: *n* = 6 Ovalbumin group: *n* = 24	4–6 weeks	Nasal lavage fluid and BAL	Control group was sacrificed at the end of the experiment: *n* = 6 mice Experimental groups were sacrificed at different time points for sample collection as follows: 1 week: *n* = 6 mice 2 weeks: *n* = 6 mice 4 weeks: *n* = 6 mice 6 weeks: *n* = 6 mice	16S rRNA gene sequencing (V3-V4 region)	OMEGA soil DNA extraction kit	Illumina MiSeq	α-diversity: Shannon index and β-diversity: Weighted UniFrac distance	QIIME 2	Upper airway microbiome of the OVA induced mice had significantly higher α-diversity than control mice. Insignificant α-diversity difference in the lower airway microbiome of the OVA induced mice and control mice. Significant difference detected in β-diversity between the OVA-induced mice and control mice. The dominant respiratory microbiome in the acute inflammatory and airway remodelling stages were different. Acute inflammatory stage: ↑ Relative abundance of *Pseudomonas* spp. Airway remodelling stage: ↑ Relative abundance of *Staphylococcus* spp. and *Cupriavidus* spp.
Lower airway microbiome											
[48] ‘High-throughput 16S rDNA sequencing of the pulmonary microbiome of rats with allergic asthma’	China	Normal control group: *n* = 4 Saline control group: *n* = 4 Allergic asthma group: *n* = 4	4–6 weeks	Lung tissues	1 time point Normal control group: lung tissues on day 0 Saline control and allergic asthma groups: lung tissues on day 29	16S rRNA gene sequencing (V4−V5 region)	No mention	Illumina high-throughput technology (Illumina PE250)	α-diversity: Chao index, coverage index, Shannon index, and Simpson index and β-diversity: Bray–Curtis	Mothur	The α-diversity of the lower airway microbiome in the allergic asthma group increased. Significant difference between normal control group and allergic asthma group was detected. Normal control group: ↑ Proteobacteria. Allergic asthma group: ↑ Firmicutes.
Lower airway and gut microbiome											
[49] ‘Alteration of Lung and Gut Microbiota in IL-13-Transgenic Mice Simulating Chronic Asthma’.	Korea	IL-13 overexpressing transgenic (TG) mice: *n* = 30 C57BL/6 wild-type (WT) mice: *n* = 30	10-week-old mice for both groups	BAL, lung tissue and faecal	1 time point	16S rRNA gene sequencing (no mention of region)	FastDNA SPIN Kit	Illumina MiSeq	α-diversity: Shannon index, Chao1 index, and the Inverse Simpson’s diversity index and β-diversity: Weighted UniFrac distances	QIIME	No significant difference in α-diversity was observed. Altered β-diversity in lung and gut microbiota in the IL-13 TG mice compared to the WT mice. IL-13 TG mice (lungs): ↑ Proportion of Proteobacteria and Cyanobacteria. ↓ Amount of Bacteroidetes IL-13 TG mice (gut): ↓ Firmicutes and Proteobacteria.

## Data Availability

The data presented in this study are available on request from the corresponding author.

## Supplementary Materials

The following supporting information can be downloaded at: https://www.mdpi.com/article/10.3390/microorganisms11040939/s1, File S1: search strategy; Table S1: data collection form; Table S2: quality assessment tools.
