# Supplementary material for: Paediatric Asthma and the Microbiome: A Systematic Review
Source: Microorganisms. 2023 Apr 3;11(4):939. doi: 10.3390/microorganisms11040939 (PMC10142236; doi:10.3390/microorganisms11040939)
Supplement: Supplementary file 1 [file microorganisms-11-00939-s001.zip › microorganisms-2260329-supplementary.pdf]

**File S1 search strategy**

**PubMed – up to February 2022**

(("asthma"[MeSH Terms] AND ("microbiome s"[All Fields] OR "microbiomic"[All Fields] OR "microbiomics"[All Fields] OR "microbiota"[MeSH Terms] OR "microbiota"[All Fields] OR "microbiome"[All Fields] OR "microbiomes"[All Fields] OR ("microbiota"[MeSH Terms] OR "microbiota"[All Fields] OR "microbiotas"[All Fields] OR "microbiota s"[All Fields] OR "microbiota"[All Fields]) OR "dysbiosis"[MeSH Terms])) AND ((English[Filter]) AND (all child[Filter] OR newborn[Filter] OR all infant[Filter] OR infant[Filter] OR preschool child[Filter] OR child[Filter] OR adolescent[Filter])) AND ((English[Filter]) AND (all child[Filter] OR newborn[Filter] OR all infant[Filter] OR infant[Filter] OR preschool child[Filter] OR child[Filter] OR adolescent[Filter]))) NOT (review[Filter])

**EBSCO – up to February 2022**

1. Asthma (abstract)
2. microbiome OR dysbiosis OR microbiota (abstract)
3. S1 AND S2
4. Limit S3 to language: English
5. Limit S4 to Age: all child (0-18 years)
6. Limit S5 to Source Types: Academic journals
7. review (Title)
8. S6 NOT S7

**Web of science – up to February 2022**

1. TOPIC: (asthma)

Indexes=SCI-EXPANDED, SSCI, A&HCI, CPCI-S, CPCI-SSH, ESCI Timespan=All years

2. TOPIC: (microbiome)

Indexes=SCI-EXPANDED, SSCI, A&HCI, CPCI-S, CPCI-SSH, ESCI Timespan=All years

3. TOPIC: (microbiota)

Indexes=SCI-EXPANDED, SSCI, A&HCI, CPCI-S, CPCI-SSH, ESCI Timespan=All years

4. TOPIC: (dysbiosis)

Indexes=SCI-EXPANDED, SSCI, A&HCI, CPCI-S, CPCI-SSH, ESCI Timespan=All years

5. #2 OR #3 OR #4

6. #1 AND #5

7. #6 NOT adult

8. #7 NOT review

7. Refined by: English

**Science Direct – up to February 2022**

1. TOPIC: (asthma)

2. TOPIC: (microbiome)

3. TOPIC: (microbiota)

4. TOPIC: (dysbiosis)

5. #2 OR #3 OR #4

6. #1 AND #5

7. #6 NOT adult

7. Refined by: original research

**Table S1:** data collection form which was designed to address objectives of the current systematic review. The identified studies were grouped based on their microbiome environment whether upper, lower airway or gut microbiome.

| Citation and title of the article | Country | Study design | Sample size | Age | Sample collected | Time of sample collection | Microbiome detection method | Genomic DNA extraction | Sequencing platform | Microbiome diversity assessment | Bioinformatics pipeline | Findings |
|---|---|---|---|---|---|---|---|---|---|---|---|---|
| **Upper airway microbiome** | | | | | | | | | | | | |
| | | | | | | | | | | | | |
| | | | | | | | | | | | | |
| **Lower airway microbiome** | | | | | | | | | | | | |
| | | | | | | | | | | | | |
| | | | | | | | | | | | | |
| **Upper and lower airway microbiome** | | | | | | | | | | | | |
| | | | | | | | | | | | | |
| | | | | | | | | | | | | |
| **Upper airway and gut microbiome** | | | | | | | | | | | | |
| | | | | | | | | | | | | |
| | | | | | | | | | | | | |
| **Lower airway and gut microbiome** | | | | | | | | | | | | |
| | | | | | | | | | | | | |
| | | | | | | | | | | | | |
| **Upper, lower airway and gut microbiome** | | | | | | | | | | | | |
| | | | | | | | | | | | | |
| | | | | | | | | | | | | |
| **Gut microbiome** | | | | | | | | | | | | |
| | | | | | | | | | | | | |
| | | | | | | | | | | | | |
