# Supplementary material for: Paediatric Asthma and the Microbiome: A Systematic Review
Source: Microorganisms. 2023 Apr 3;11(4):939. doi: 10.3390/microorganisms11040939 (PMC10142236; doi:10.3390/microorganisms11040939)
Supplement: Supplementary file 1 [file microorganisms-11-00939-s001.zip › Table S2 quality assessment tools.pdf]

Table S2 quality assessment tools

Risk of bias assessment tool 1 Newcastle–Ottawa Scale (NOS) for human case-control studies.

Citation	Selection[1]				Comparability[2]	Exposure[3]			Total[4]	Rate[5]
	Is the case (asthma) definition adequate?	Representativeness of the cases	Selection of controls	Definition of controls	Comparability of cases and controls on the basis of the design or analysis	Ascertainment of exposure	Same method of ascertainment for cases and controls	Non-response rate		

[1]A study can be awarded a maximum of one star for each numbered item within the selection, the star was translated into 1, if no star, then 0.

[2]A maximum of two stars can be given for comparability, the star was translated into 2 or 1, if no stars then 0.

[3]A study can be awarded a maximum of one star for each numbered item within the exposure, the star was translated into 1, if no star, then 0.

[4]The maximum number was nine stars, which were translated into nine.

[5]Quality rate was identified as good, fair or poor based on Agency for Health Research and Quality standards.

Risk of bias assessment tool 2 Newcastle–Ottawa Scale (NOS) for human cohort studies.

Citation	Selection[1]				Comparability[2]	Outcome[3]			Total[4]	Rate[5]
	Representativeness of the exposed cohort	Selection of the non-exposed cohort	Ascertainment of exposure to implants	Demonstration that outcome of interest (asthma) was not present at start of study	Comparability of cohorts on the basis of the design or analysis	Assessment of outcome	Was follow up long enough for outcome to occur	Adequacy of follow-up of cohorts		

[1]A study can be awarded a maximum of one star for each numbered item within the selection, the star was translated into 1, if no star, then 0.

[2]A maximum of two stars can be given for comparability, the star was translated into 2 or 1, if no stars then 0.

[3]A study can be awarded a maximum of one star for each numbered item within the outcome, the star was translated into 1, if no star, then 0.

[4]The maximum number was nine stars, which were translated into nine.

[5]Quality rate was identified as good, fair, or poor based on Agency for Health Research and Quality standards.

Risk of bias assessment tool 3 Newcastle–Ottawa Scale (NOS) for human cross-sectional studies.

Citation	Selection[1]				Comparability[2]	Outcome[3]		Total	Rate[5]
	Representativeness of the sample	Sample size	Non-respondents	Ascertainment of the exposure	The subjects in different outcome groups are comparable, based on the study design or analysis. Confounding factors are controlled.	Assessment of outcome	Statistical test		

[1]Apart from 'ascertainment of the exposure' item, a study can be awarded a maximum of one star for each numbered item within the selection, the star was translated into 1, if no star, then 0. The ascertainment of the exposure item can be awarded two stars if validated measurement tool was used in the study.

[2]A maximum of two stars can be given for comparability, the star was translated into 2 or 1, if no stars then 0.

[3]A study can be awarded a maximum of two stars for assessment of outcome if independent blind assessment was used in the study. While statistical test can be awarded a maximum of one star. The star was translated into 1 or 2 if no star, then 0.

[4]The maximum number was ten stars, which were translated into 10.

[5]Quality rate was identified as good, fair or poor based on Agency for Health Research and Quality standards.

Risk of bias assessment tool 4 The systematic review centre for laboratory animal experimentation risk of bias assessment tool for mouse model studies.

Citation	Selection bias			Performance bias		Detection bias		Attrition bias	Reporting bias	Other
	Sequence generation	Baseline characteristics	Allocation concealment	Random housing	Blinding	Random outcome assessment	Blinding	Incomplete outcome data	Selective outcome reporting	Was the study apparently free of other problems that could result in high risk of bias?
